# Neurobiological Correlates in Forensic Assessment: A Systematic Review

**DOI:** 10.1371/journal.pone.0110672

**Published:** 2014-10-20

**Authors:** Toon van der Gronde, Maaike Kempes, Carla van El, Thomas Rinne, Toine Pieters

**Affiliations:** 1 Department of Pharmacoepidemiology and Clinical Pharmacology, Utrecht Institute for Pharmaceutical Sciences (UIPS), and Freudenthal Institute, Utrecht University, Utrecht, the Netherlands; 2 Netherlands Institute of Forensic Psychiatry and Psychology, Pieter Baan Center, Forensic Psychiatric Observation Clinic, Utrecht, the Netherlands; 3 Section Community Genetics, Department of Clinical Genetics and EMGO+, VU University Medical Centre, Amsterdam, the Netherlands; University of Medicine & Dentistry of NJ - New Jersey Medical School, United States of America

## Abstract

**Background:**

With the increased knowledge of biological risk factors, interest in including this information in forensic assessments is growing. Currently, forensic assessments are predominantly focused on psychosocial factors. A better understanding of the neurobiology of violent criminal behaviour and biological risk factors could improve forensic assessments.

**Objective:**

To provide an overview of the current evidence about biological risk factors that predispose people to antisocial and violent behaviour, and determine its usefulness in forensic assessment.

**Methods:**

A systematic literature search was conducted using articles from PsycINFO, Embase and Pubmed published between 2000 and 2013.

**Results:**

This review shows that much research on the relationship between genetic predisposition and neurobiological alterations with aggression is performed on psychiatric patients or normal populations. However, the number of studies comparing offenders is limited. There is still a great need to understand how genetic and neurobiological alterations and/or deficits are related to violent behaviour, specifically criminality. Most studies focus on only one of the genetic or neurobiological fields related to antisocial and/or violent behaviour. To reliably correlate the findings of these fields, a standardization of methodology is urgently needed.

**Conclusion:**

Findings from the current review suggest that violent aggression, like all forms of human behaviour, both develops under specific genetic and environmental conditions, and requires interplay between these conditions. Violence should be considered as the end product of a chain of life events, during which risks accumulate and potentially reinforce each other, displaying or triggering a specific situation. This systematic review did not find evidence of predispositions or neurobiological alterations that solely explain antisocial or violent behaviour. With better designed studies, more correlation between diverse fields, and more standardisation, it might be possible to elucidate underlying mechanisms. Thus, we advocate maintaining the current case-by-case differentiated approach to evidence-based forensic assessment.

## Introduction

Violent crime is a complex problem without simple solutions. Given the prevalence of violent criminality in our society, [1]–[3] an understanding of the predictive and causal factors of violence is needed to improve assessment of criminal responsibility, risk assessment and management practices. What factors put individuals at risk for developing violent behaviour and committing a crime? What factors promote resiliency and protect individuals from re-offense? In the last 50 years, much has been learned about psychosocial risk factors that predispose people to violence. [4], [5] However, psychosocial and biological causes of crime are inseparably entwined and are constantly interacting. Over the past two decades, on the tails of the genome project and a revolution in brain imaging, scientists across the world have tried to solve the enormous jigsaw puzzle of the biology of violent and criminal behaviour. These efforts have advanced our knowledge about and understanding of the biological factors and mechanisms involved in violent and criminal behaviour. [6], [7] Nevertheless, forensic (risk) assessment is still mainly based on psychosocial risk factors. [8]–[10] The challenge now is to integrate these innovative neurobiological and genetic findings with current criminal assessment practices. When an individual suffers from a severe mental disorder that leads to a crime, it is generally agreed in most jurisdictions that he or she cannot be held criminally responsible and should be exempt from penal consequences. [7], [11]–[13] Psychiatrists and psychologists are often called upon to render the expert opinions needed for legal determinations of criminal responsibility and risk for recidivism [14], [15].

Currently, forensic assessment is predominantly focused on psychosocial factors, however, till date, risk assessment instruments do not include biological risk factors. [8]–[10] We do know that psychosocial factors interact with biological factors in shaping (violent) behaviour. [6] Research shows that a small proportion of offenders, approximately 6%, account for the majority of all crimes [16] and that 5% of families account for more than 50% of all arrests. [17] With the increased knowledge of biological risk factors, interest is growing to include (more) information about biological risk factors in forensic assessments. In recent years, neuroscientific evidence, e.g. neurogenetics [18] and neuroimaging, has begun to be used to document a person's tendency towards aggression as was done in the case of the serial killers Brian Dugan [19] and Bradley Waldroup [20] and two recent murder cases in Italy. [11], [20], [21] This may yield several benefits. First, biological risk factors would lead to more objective measures of criminal responsibility or risk assessment of violent behaviour since they are thought to be less prone to manipulation. Second, assessing biological risk factors may reveal new information that could not be previously determined, such as assessing the possible role of a specific brain damage in criminal behaviour. Third, assessing information on biological risk factors would provide more information on the interaction between social and biological risk factors and their relationship with violent behaviour.

In summary, a better understanding of the neurobiology of violent criminal behaviour would help to provide insight into whether and how assessment of biological risk factors could improve forensic assessment. This systematic review aims to provide an overview of the current evidence about biological risk factors that predispose people to antisocial and violent behaviour and determine its usefulness in forensic assessment. As a framework to review the available literature, we adopt a biosocial model of violence as used by Raine. [6] Thus, we focus on evidence from genetics and interaction with pre- and post-natal environments, as well as related areas such as neuroanatomy, neuropsychology and neurology, neurophysiology, neurochemistry and endocrinology. This multi-disciplinary approach offers additional insight into the criminal mind and the underlying causes of violent behaviour to assist in forensic assessment.

Our hypothesis is that a general model that holds on a population level is, as of now, not evidence based. Forensic assessment remains to be done on a case-by-case base. Though several pieces of knowledge can be connected, an overall picture that leads to a general understanding of criminal violence is not yet possible.

First, we present brain (dys-)functioning and behavioural effects with a special focus on brain anatomy and neurotransmitters. Subsequently, we review genetic and environmental influences on antisocial behaviour, as well as possible correlations with risk factors. The relevant models and theories for each section will be discussed, including supporting evidence for each one. Finally, we consider the possible implications for forensic assessment and address research challenges.

## Method

### Data sources

Two literature searches were conducted in the electronic databases of PsycINFO, Embase and Pubmed. The first search concerned reviews and/or meta-analyses published between 2000 and 2013; the second search retrieved empirical research published between 2010 and 2013. We selected publications by using a query based on keywords concerning criminality, aggression, antisocial behaviour or psychopathy in combination with either neurosciences or genetics. The exact query is provided in the appendix.

#### Inclusion

In both searches we included publications using the following criteria: a) published in peer-reviewed journals, b) written in English, and c) included offender populations. We excluded papers that were: a) case reports, books, conference abstracts, letters, b) written in languages other than English, c) published before the dates mentioned, d) animal studies, or e) concerned only paedophilia/paraphilia due to the likeliness of these being caused by mechanisms other than violent criminality.

#### Selection

**Figure 1:**
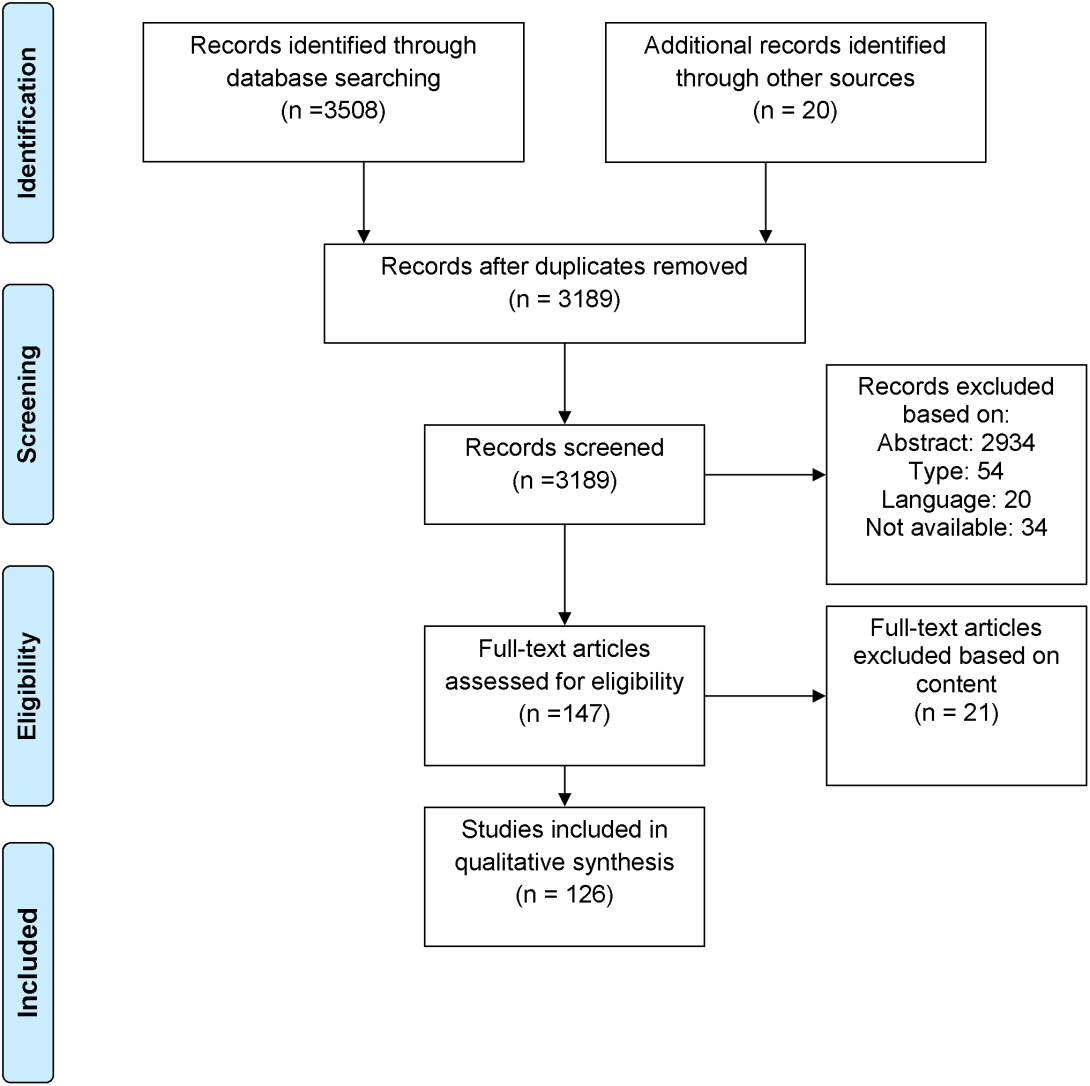
Selection of publications. Out of the 3508 found articles, 126 were used for this article.

All selected publications were assessed for relevance based on both title and abstract. The articles were judged for inclusion by two independent researchers based on content. The reviews and/or meta-analyses were divided into two groups. The first group of articles related to a criminal or forensic context. The second group of articles addressed types of behaviour, i.e. physical aggression and violence that are most relevant to the criminal justice system in terms of personal damage for the victim and serious legal consequences for the perpetrator. Twenty articles that were deemed essential but were not found in the systematic search were added. This is shown in figure 1.

## Results

### Brain and criminology

Various disciplines study brain functioning and the effect dysfunction has on behaviour, but each has a distinct perspective and aim. The following section aims to correlate the findings across these research fields to facilitate a more thorough understanding of the possible relationships between criminal behaviour, neuroanatomy, brain biochemistry and neuropsychology.

We first present neuroanatomy, including the morphological structures of the brain that are found to be relevant in imaging studies. Next, we discuss forensic neuropsychology – this encompasses the integration of psychological findings with neurology by performing tests that specifically target an area of the brain. [9], [22] Finally, an overview of the related neurotransmitters and hormones is presented. A schematic summary of the evidence from the different research fields can be found in a series of six tables.

### Neuroanatomy

One of the challenges of neurocriminology is to trace biological markers of sociopathy with brain imaging research. Brain-imaging techniques identify physical deformities and functional abnormalities that may predispose some individuals to violence. This has led to theories of neuroanatomical deviations and criminal behaviour. In the following paragraphs, these theories and evidence for them are discussed. However, first we provide a brief overview of the most important structures mentioned in research on violent criminal behaviour and the relationships between these structures. (See Table 1).

**Table 1:** Overview of the evidence for involved brain areas.

Brain region	Population	Method	Outcomes	Reference
Prefrontalcortex	Offenders	PET	Reduced functioning	[6], [33], [37], [51], [52]
	Violent patients	PET	Reduced activity	[36]
	Aggressive subjects	EEG	EEG abnormalities	[53]
	Forensic psychiatric patients	PET	Decreased blood flow ormetabolism	[31], [53]
	Antisocial patients	MRI	Reduced frontal greyvolume	[3], [53]
	Aggressive subjects	PET	Reduced ventrolateralactivity	[44]
	Violent offenders	PET	Reduced glucosemetabolism	[32], [55]
	Murderers	PET	Reduced glucosemetabolism	[56]
	Violent psychiatric patients	PET	Reduced glucosemetabolism	[31], [33]
	Impulsive murderers	PET	Reduced glucosemetabolism	[2]
	Violent patients	MRI	Less N-acetyl aspartate	[3], [32]
	Violent patients	MRI	Lower phosphatemetabolism	[3], [32]
Amygdala	Murderers	PET	Lowered activity	[42]
	Psychopaths	fMRI	Reduced activation	[3]
Hippocampus	Violent offenders	SPECT	Low resting blood flow	[3]
	Violent offenders	PET	Reduced metabolism	[3]
	Murderers	PET	Abnormal functioning	[6], [55]
	Psychopaths	MRI	Structurally distinct	[6]
Temporallobe	Impulsive-aggressivePersonality-disordered	MRI	20% reduction	[62]
Anteriorcingulate cortex	Offenders	fMRI	Hemodynamic activitypredicts re-arrest	[65]
Hemispheres	Several populations	Several techniques	Left dorsolateral prefrontalcortex deficits	[49]
	Several populations	Several techniques	The right orbitofrontalcortex and anterior cingulatecortex right	[49]
	Affective and predatorymurderers	PET	High right hemispheresubcortical functioning	[31]
	Affective murderers	PET	Low left, high rightprefrontal functioning	[31]
	Violent psychiatricpatients	SPECT	Increased or abnormalleft limbic activity	[33]
	Offenders who werevictims of child abuse	fMRI	Reduced right temporalcortex functioning	[6]
	Psychopathic patients	MRI	Reduced right temporalcortex volume	[52]
	Incarcerated psychopaths	fMRI	Dysfunction in the righthemisphere duringabstract processing	[56]
	Antisocial and violentsubjects	fMRI, CT,EEG, ERP	Poor right hemispherefunctioning	[32]

PET: Positron Emission Tomography, EEG: Electroencephalography, (f)MRI: (functional) Magnetic Resonance Imaging, SPECT: Single-photon emission computed tomography, ERP: event-related potentials, CT: computed tomography.

An important factor in violent criminal behaviour is emotion, or the lack of it. In general, the amygdala is involved in emotions, [23]–[27] particularly negative emotions, [27]–[29] and the recognition of fear [26], [29], [30]. The regulation of emotions is thought to be dependent mostly on the prefrontal cortex, [1], [3], [13], [27], [31]–[36] which is rich in 5-hydroxytryptamine (5-HT) type 2-receptors. [2] The prefrontal cortex inhibits the amygdala, as well as other limbic system regions, like the hippocampus (involved in memory [14]), hypothalamus, anterior cingulate cortex, insular cortex, ventral striatum, and some structures connected to those regions. [1], [2], [31], [36] The prefrontal cortex also receives information from the rest of the cortex and the limbic system [1], [37].

Another important aspect in the instigation of behaviour is processing social information in one’s environment. Signals that can indicate a threat, like posture, facial expression or screaming, are directed to the lateral nucleus of the amygdala. [2], [26], [31], [38]–[40] The signals project to the basal nuclei, where they are integrated with perceptual information originating from the orbitofrontal cortex. [2], [24], [41] This can lead to a behavioural response via the central nucleus, the hypothalamus and brainstem. [2] Thus, the orbitofrontal cortex is thought to integrate the cognitive activity of the total prefrontal cortex into the emotional limbic system. [25]–[27], [37] In this manner, the prefrontal cortex restricts impulsive, disinhibited behaviour and volatile emotions [2], [25], [26], [31].

Dysfunction or neuroanatomical deviations of one or several of the above-described structures have been shown to be related to violent criminal behaviour. However, before we describe these studies, it is important to distinguish between two types of violent aggressive behaviour. Reactive, [42]–[48] emotional [45] or impulsive [2] aggression is a reaction to events, often driven by emotion. Instrumental [42]–[45], [47], [48] or premeditated [2] aggression, however, is cold and calculated. Since reactive behaviour is influenced primarily by emotions, and the instrumental is not, distinct brain areas are expected to be involved in the various forms of aggression [2], [42], [44], [46], [48].

#### Prefrontal cortex

The frontal lobe dysfunction theory states that violent and reactive aggression is a consequence of deficits in the frontal brain, mainly the prefrontal cortex. [44], [44], [49,50] Supporting evidence for this theory comes from various research areas.

First, research conducted with PET shows reduced functioning of the prefrontal cortex in offenders, [6], [33], [37], [51], [52] and reduced activity in violent patients. [36] The association is stronger in murderers with a benign social background, than in those with a bad home background, [6] as expected based on the social push theory (which will be discussed later on). Frontal abnormalities have also been found using EEGs on aggressive subjects in various populations (like violent criminals [53]), with PET in forensic psychiatric patients [31], [53] and in an MRI study of antisocial patients. [3], [53] One study distinguished between the various regions of the prefrontal cortex, and specifically found reduced activity in the ventrolateral part of the prefrontal cortex – relevant for social behavior [54] – in aggressive subjects [44].

Second, studies on brain metabolism show that in general, a reduced glucose metabolism in the prefrontal regions can be found in violent offenders. [32], [55] Specifically, it was found that murderers [56] and violent psychiatric patients [31], [33] have a lower prefrontal cortex metabolism than controls. This finding has been replicated in impulsive murderers for whom a rise in the metabolism of subcortical regions was also found, as expected, since inhibition by the prefrontal cortex is reduced. [2] However, murderers in that same group who planned their crimes did not have a lowered metabolism in their prefrontal cortex. [2] When the findings for predatory and affective murderers were separated, it was clear that affective murderers had lower prefrontal metabolic activity than predatory murderers, who resembled controls [32], [53].

Third, a study examining N-acetyl aspartate, considered a marker of neuronal integrity, [3], [57] showed that violent patients had less N-acetyl aspartate in their prefrontal cortex compared to controls. [3], [32] More importantly, the frequency of violence was inversely correlated with the levels of N-acetyl aspartate. [3] A lower phosphate metabolism was also found in the prefrontal cortex of the violent patients [3], [32].

In general, deficits in the prefrontal area, mainly the ventromedial part, have been found to be related to poor control of reactive violence. [2], [31], [45], [46], [48] Instrumental violence, on the other hand, is thought to be associated with the dysfunction of both the ventromedial prefrontal cortex and the amygdala. [46] In addition, individual differences appear to be important. Personal differences in the ability to modulate emotions have been shown to be linked to prefrontal activation. [2] This might be relevant in understanding vulnerability to violence and aggression [2].

#### Amygdala

The amygdala is comprised of thirteen nuclei, together forming one structure. [54] The integrated emotion systems model hypothesises that deviant social behaviour, such as violence, is the result of inhibited emotional development caused by amygdala dysfunction. [28], [35], [58]–[60] This is supported by a study in murderers, that showed a lowered amygdala activity, compared to age- and sex-matched controls [42].

Moreover, the integrated emotion systems model states that amygdala damage leads to impaired interpretation of emotions. [23], [61] This results in diminished empathy, failure to recognise fearful expressions and impaired passive avoidance learning, all of which have been documented in psychopaths [25], [58].

These studies lead to the conclusion that prefrontal cortex function [3], [53] or size [1] and amygdala function [3] are related to violent aggression. It is possible that both the frontal lobe dysfunction theory and the integrated emotion systems model are true, and reinforce each other. It is also possible that the former theory explains reactive aggression and the latter explains instrumental aggression.

#### Hippocampus, temporal lobe, anterior cingulate cortex

In addition to the amygdala and prefrontal cortex, other brain structures have also been shown to differ in criminal subjects compared to the general population. The hippocampus is part of the limbic system and is involved in memory. The hippocampus has been shown to function abnormally in violent offenders [3] and subjects who commit murder [6], [55] and is structurally distinct in psychopaths [6].

The temporal lobe contains the hippocampus and plays a key role in the formation of explicit long-term memory that is modulated by the amygdala. A 20% reduction of the temporal lobe was found in aggressive psychopaths, [62] and functional abnormalities of the temporal lobe were found in violent psychiatric patients. [33] Sexual offending might also originate in the temporal lobe. [37], [63], [64] However, this goes beyond the scope of this review.

The anterior cingulate cortex is a limbic region involved in response selection, behavioural regulation, inhibition, [65], [66] and empathy. [26] Interestingly, anterior cingulate cortex hemodynamic activity predicts re-arrest; higher activity leads to better inhibitory control and recurrence rates that are half of those with low activity. [65] If this correlation is replicated, it could possibly lead to a better tool for risk analysis in combination with known psychosocial risk factors, as scepticism remains regarding the sensitivity and specificity of emerging neurobiological markers as independent tools [61].

#### Hemispheres

Apart from findings concerning specific brain areas, much research is directed at structural dysfunction in the left or right hemispheres. The left Hemisphere Activation Hypothesis states that psychopaths have problems shifting from left hemisphere activity to right hemisphere activity, and specifically processing information in the left hemisphere. [49] Support for this hypothesis is offered by deficits in the left dorsolateral prefrontal cortex, [49] which is associated with attentional control. [27] This makes sustaining attention in the left hemisphere more difficult. [49] Deficits in the right orbitofrontal cortex and anterior cingulate cortex support this since they are involved in the ability to change to right hemisphere activity [49].

In one study, both affective and predatory murderers had higher subcortical right hemisphere functioning than controls, but affective murderers also had lower left and higher right prefrontal functioning. [31] In another study, violent psychiatric patients were found to have increased or abnormal left limbic activity. [33] Offenders who were victims of child abuse have been shown to have reduced right temporal cortex functioning, [6] which is associated with conduct disorder. [26] Reduced volume of the right temporal cortex has been found in psychopathic patients [52].

General deficits in the right hemisphere have been proposed as well. During abstract processing, which is thought to be based in the right temporal lobe, a dysfunction in the right hemisphere was found in incarcerated psychopaths. [56] In antisocial and violent populations, poor right hemisphere functioning has also been observed [32].

Overall, several brain areas appear to be deficient in violent individuals. An overview of findings is given in table 1. However, it is unknown whether these deficits always result in violent behaviour per se, since this was not investigated in the studies mentioned. The evidence that brain deficits are related to violent behaviour is mainly based on case reports such as the one on Phineas Gage (this case will be discussed in more detail later on), which have been shown to be untrue or only partly true. [21] Moreover, case reports that show no violent behaviour in persons with brain deficits are also available. [21] Therefore, more research should be done on how brain deficits or alterations are related to the actual instigation of violent behaviour. In this respect, it may be more fruitful to examine how altered brain *function* is related to violent behaviour.

### Neuropsychology and neurology

The brain areas described above have distinct functions. In the following paragraphs, we describe additional evidence for a relationship between altered brain *function* and the propensity for violent behaviour. A schematic overview of the evidence is given in table 2.

**Table 2:** Overview of the evidence for brain functions.

Brain function	Population	Method	Outcomes	Reference
Executivefunctioning	Boys with apaternal historyof substanceabuse	Neuropsychologicaltests	Low executive functioningcan predict aggressivebehaviour	[53]
Psychophysiology	Aggressive anti-socials andpsychopaths	Resting heart rate andskin conductance	Low autonomicarousal in rest	[41], [45], [52], [55]
	Children	Resting heart rate andskin conductance	Low autonomic arousal ispredictive for becomingoffenders	[32], [55], [66]

#### Executive functioning

As shown above, murderers’ prefrontal cortices often seem to be an affected brain area. Numerous studies have demonstrated that the prefrontal cortex is important in executive functioning [1], [6], [32], [37], [43], [50], [53], [60] (organising cognitive processes), to attain a future goal. [1], [67] Executive functioning includes attention control, behavioural flexibility, working memory, self-awareness, abstract decision making and planning. [6], [14], [43], [63], [68] Executive functioning is required for much complex behaviour, such as social functioning and managing competing interests, [67] and can be measured using neuropsychological tests [53], [68].

Lesions in or dysfunction of the prefrontal cortex [37] or the frontal lobe in general [51] lead to impaired executive functioning. Impaired executive functioning is associated with antisocial [6], [68] and aggressive [51], [53] behaviour. More importantly, low executive functioning can predict aggressive behaviour in boys with a paternal history of substrance abuse. [53] This could possibly help determine the risk for recidivism in general [53].

#### Inhibition

Another structure that has been shown to be significant in violent behaviour is the anterior cingulate cortex. The anterior cingulate cortex [65] and the serotonergic neurons in the prefrontal cortex are thought to be important for behavioural inhibition. [37] Indeed, prefrontal damage, especially orbital damage, [8], [37], [68] does lead to lower inhibition and pseudopsychopathic behaviour [8], [69].

#### Empathy

Several brain regions are involved in the instigation of empathy. Lesions in the orbitofrontal cortex [53], prefrontal cortex, [41] amygdala [41], [54] or anterior cingulated cortex [41] are related to a lack of empathy. A lack of empathy is indeed often found in offenders. [37], [70] These abnormalities in the emotional regulation circuitry are thought to lead to reactive aggression and the violence seen in these individuals [2].

#### Psychophysiology

Differences in physiology between the offending and general population have been found, long before brain-imaging techniques existed. In this respect, one of the most replicated observations in aggressive anti-socials and psychopaths is low autonomic arousal in rest, measured by resting heart rate and skin conductance [41], [45], [52], [55].

It is likely that low autonomic arousal is related to or a result of anatomical and functional deviances in violent offenders. For example, it is proposed that reduced noradrenergic functioning and reduced right hemisphere functioning would explain the low autonomic arousal found in violent criminals. [32] Moreover, autonomic arousal is also controlled by the amygdala, [28] which has been found to be less functional in murderers. Therefore, low arousal may be a marker for amygdala dysfunction [39].

Although the origin of low autonomic arousal is of interest, forensic risk assessment would particularly benefit from knowledge about how low autonomic arousal may be related to or predict (violent) criminal behaviour. Several theories are of interest.

First is the fearlessness theory, which states that low levels of arousal are a marker for low levels of fear. [3], [6], [32], [52], [71] Fearlessness predisposes a person to criminal behaviour, because criminality requires low fear levels. [6], [32], [71] Also, the effectiveness of learning through conditioning is diminished by less anticipatory fear for punishment, [32], [44], [52], [58], [62], [71], [72] leading to impaired socialisation. [6], [42], [58], [71] Second, the stimulation-seeking theory states that low arousal will make subjects seek more exciting, possibly criminal activities, trying to relieve their boredom [32], [42], [52], [55], [58], [71], [72].

In accordance with the notion that low autonomic arousal may not only be related to but may also predict criminal behaviour, several studies have shown a link with future criminal offenses, [32], [52], [55], [72], [73] aggression [6], [32], [55], [62], [71], [72] – especially instrumental aggression [42] – and antisocial behaviour. [6], [32], [41], [55], [58], [62] It has even been shown that low autonomic arousal is predictive of children growing up to become offenders. [32], [55], [66] In one study, aggressive children had lower heart rates than nonaggressive children (p<0.001), and children with lower heart rates were rated as aggressive more often than those with high heart rates (p<0.003). [71] Therefore, autonomic arousal may be an interesting marker to improve risk assessment in future. A downside to using autonomic arousal as a marker is that as of yet it is unknown what the cut-off point for increased risk would be.

### Neurotransmitters, hormones, and toxins

Neurotransmitters and some hormones are important for communication between neurons in the brain and thus, they are of importance in the instigation of behaviour. Therefore, researchers have sought the origin of criminal behaviour in a disturbed balance between some of these neurotransmitters or hormones. Since toxins influence the levels of these neurotransmitters, they could also be of significance. A schematic overview of the evidence is provided in table 3.

**Table 3:** Overview of the evidence for involved neurotransmitters and hormones.

Neurotransmittersandhormones	Population	Method	Outcomes	Reference
Serotonin	Several	Several	Low levels of serotonin areassociated with bothreactive and instrumentalaggression	[2], [7], [8], [10], [25], [31], [34], [41], [43], [45], [51], [66], [74]–[78]
	Several	Several	Low levels of serotonin areassociated with impulsivity	[7], [10], [22], [25], [31], [34], [37], [43], [51], [72], [77], [79]
	Boys withconduct-disorderandrecidivists	5-hydroxyindoleaceticacid levelmeasurement	Predict aggression two tothree years in the future	[2]
Noradrenalin	Humans	Plasma andcerebrospinalfluidmeasurements,report scale	Noradrenalin is positivelycorrelated with impulsivity	[31]
	Humans	Drugadministering	Increases in affectiveaggression whennoradrenalin is elevated	[45]
Dopamine	Humans,offenders	Gene expression	Activation of D2, D3 andD4-receptors are related toaggressive impulses	[7], [87], [88]
GABA	Humans	Benzodiazepine use	Benzodiazepines, areeffective in reducingaggression	[45], [91]
Cortisol	Boys,adolescentsand adults	Salivameasurement	Low cortisol levels wereassociated with aggressivebehaviour	[10], [33], [39], [41], [43], [55], [55], [72], [73,93,93,93]
Testosterone	Children,adults	Plasmatestosterone	Delinquency	[6], [93]
	Offenders	Salivameasurement	Antisocial behaviour	[64]
	Males	Plasma testosterone	Aggression	[2], [6], [10], [33], [55], [66], [72], [93], [95]
	Several	Several	Dominance	[25]
	Hypogonadaladolescents	Testosteroneadministration	More physical aggression	[93]
Thyroid hormones	Delinquent boys	Serum levels	Relationship between T3and antisocial behaviour	[41]
	Formerjuveniledelinquents	Serum levels	T3 levels correlate withpersistent criminalbehaviour	[41]

GABA : γ-aminobutyric acid, T3: triodothyronine.

#### Serotonin

One of the most replicated findings is the relationship between serotonin and aggression. Numerous studies have shown that low levels of serotonin are associated with both reactive and instrumental aggression [2], [7], [8], [10], [25], [31], [34], [41], [43], [45], [51], [66], [74]–[78] and impulsivity. [7], [10], [22], [25], [31], [34], [37], [43], [51], [72], [77], [79] In addition, low serotonin levels [31], [55] and reduced levels of 5-hydroxyindoleacetic acid, a serotonin metabolite, have been found in aggressive or violent populations. [2], [31], [41], [43], [52], [74] Furthermore, a negative correlation between the serotonin 5-HT1A receptor and aggressive behaviour has been established. [79] In impulsive aggressive subjects, reduced serotonin transporter availability was found in the anterior cingulated cortex. [78] Moreover, one study showed that low levels of serotonin predicts recidivism. [7] 5-hydroxyindoleacetic acid levels have been found to predict aggression two to three years in the future in boys with conduct-disorder and recidivists. [2] Antidepressant drugs that act on serotonin, like SSRI’s that cause serotonin levels to go up, can reduce violent behaviour in some individuals, [10], [33], [45], [80], [81] especially those with high impulsive aggressiveness. [48] Although the above-mentioned studies show that aggression and violence are related to low levels of serotonin, other results seem to indicate the opposite. Metabolic enzymes such as monoamine oxidase A (MAO-A) also contribute to aggression because they function to alter neurotransmitter levels. Since MAO-A catalyses the deamination of serotonin, reduced MAO-A activity will lead to higher levels of this neurotransmitter. [57], [66], [74], [82], [83] However, MAO-A deficiency, resulting in higher levels of serotonin, has been shown to increase reactive aggression [20], [74], [84] and low activity to increase criminal behaviour. [7], [84] This is called the serotonin paradox. One author argues that the change in behaviour due to MAO-A is actually a consequence of secondary effects, and cannot be explained by its effect on neurotransmitters alone. [74] Taken together, the above-described results do show that the relationship between serotonin and aggressive or violent behaviour is more complicated than is sometimes presented in the courtroom. [81] An individual risk-assessment on the basis of serotonin levels is not supported by evidence.

#### Noradrenalin

Although the relationship between serotonin and aggression and violent behaviour seems strong, there is also evidence that other neurotransmitters are involved. For example, noradrenalin levels, a neurotransmitter involved in the inhibition of memory storage and experiences, [48] in plasma and cerebrospinal fluid are positively correlated with impulsivity [31] and affective aggression. [45] This does not provide much information about the exact site of noradrenalin release, but makes drugs counteracting noradrenergic function interesting for preventing aggressive behaviour [31], [45].

#### Dopamine

Dopamine levels, a neurotransmitter important for rewards, delayed rewards and risk taking, [85] have been correlated with violent [86]–[88] and antisocial behavior [82], [89] and sensation seeking. [85], [90] Activation of dopamine receptors, especially the D_2_, [88] D_3_ [7] and D_4_ [87] receptors, are related to aggressive impulses, [2], [25], [45] and regulated by serotonin. [25] D_2_ receptor agonists have successfully been used to treat aggression in some patient groups, especially those who are psychotic [48], [76].

#### GABA

Finally, another neurotransmitter, γ-aminobutyric acid (GABA), also seems to inhibit aggression. [8], [45], [48] Indeed benzodiazepines, substances that enhance GABA signalling, are effective in reducing aggression in humans, [45], [91] though in specific subsets it increased aggressive behaviour [48].

In summary although evidence for the effects of the serotonin system on violent aggression is strongest, several other neurotransmitters seem to affect aggression and violence. To make the situation even more complicated, the neurotransmitter system also has interactions with other systems in the body, such as the endocrine system.

#### Hormones

One of the most studied relationships is that between the stress system and aggression. Since the prefrontal cortex contains some of the highest levels of cortisol receptors of the primate brain, low levels of stress hormone will alter the turnover of various neurotransmitters. [1] Adrenocorticotropic hormone (ACTH) is produced when cortisol is suppressed, and it increases serotonin metabolism. [31] This results in lower serotonin levels. [31] Cortisol itself seems to be inversely correlated to levels of serotonin. [92] As such, low cortisol levels were associated with sensation seeking [41] and decreased sensitivity to punishment [39], but also with aggressive behaviour in boys, [10], [41], [43], [55], [93] adolescents [39], [55], [93] and adults [33], [72], [73], [93]. However, similar to the serotonin system, these relationships are not unequivocal since high levels of stress hormone have also been found to be related to aggressive behaviour. [94] The key seems to be that the production of cortisol is deregulated.

A second important hormone in relation to violent aggression is testosterone. Plasma testosterone levels have been associated with childhood and adult delinquency, [6], [93] antisocial behaviour, [64] aggression [2], [6], [10], [33], [55], [66], [72], [93], [95] and dominance, [25] but a correlation with social success has also been suggested. [93] These correlations have not always been well replicated. [93] The effect of testosterone on aggression is not visible in young children, possibly because aggression in childhood does not increase dominance as it does in adulthood. [6] In 9–11 year old boys, the association between testosterone and aggression has been documented [55].

During development, testosterone induces or inhibits cell death, guiding the brain to typical male pathways. [43], [74], [93] Later, it stimulates neural pathways associated with aggression. [74] Testosterone receptors have in fact been found throughout the limbic system. [43] The association between testosterone and aggression has been confirmed by users of anabolic steroids. [74] In tests, testosterone injections led to a shift in sensitivity from punishment to reward. [39] Hypogonadal adolescents receiving testosterone became more aggressive physically, but not verbally. [93] This could be explained by changes in musculature as well [93].

Testosterone and cortisol inhibit each other’s production. [25], [39], [58] This means it is possible that the findings of the effects of cortisol are actually due to testosterone, or the other way around. The triple balance of emotion model states that the hyposensitivity for punishment and the hypersensitivity for reward found in psychopaths [40] could be explained by a high testosterone-to-cortisol ratio. [25], [39], [52], [58] Indeed, testosterone increases sensitivity to reward, [25], [39] and low cortisol stimulates the hypothalamic–pituitary–gonadal axis, reducing sensitivity to fear [39], [58].

A third group of hormones related to antisocial behaviour are the thyroid hormones. T_3_ and T_4_ have been related to antisocial behaviour. [41] T_3_ has also been specifically linked to recidivism [41].

#### Other substances

Both the endocrine and neurotransmitter system are influenced by substances in the body other than hormones. For example, the connection between alcohol and violence is well documented. [34], [91], [96] Over half of all violent crimes occur under the influence of alcohol. [91] Alcohol’s mechanism of action is thought to be dependent on the function of GABA_A_, [48] 5-HT and N-methyl-D-aspartate receptor (NMDA)-receptors. [91] A lowering of tryptophan, thought to be parallel to the level of brain serotonin, has been documented two hours after alcohol consumption in a normal population [34].

In addition, alcohol inhibits the capacity of the prefrontal cortex, leading to impaired executive functioning. [1], [97] This makes it a disinhibiting factor, leading to acting out what was previously inhibited. [10], [70], [92], [97] Alcohol is an aggravating factor in domestic violence, [33], [51] and increases the chances of committing homicide [98].

Abuse of other substances also increases risk of violence. [4], [8], [33], [50], [51], [99], [100] Cocaine for example enhances dopamine signalling, [1] and decreases the capacity to control impulses [8], [70].

Another substance that may affect neurotransmitter levels is cholesterol. Low cholesterol has been linked to aggression. [2], [55] In community samples of psychiatric patients or criminal offenders with low cholesterol levels, an increase in violence was found. [55] A possible explanation for this observation is that low cholesterol leads to lower serotonin levels [55].

To summarize, the various neurotransmitter systems in the central nervous system have complex interactions with each other and with other systems in the body such as hormones and toxins. This makes it hard to understand how aggression and violence are regulated in individuals.

### Genetic and environmental influences

#### Genetics, GxE

Genetic influences on antisocial and aggressive behaviour have been documented in literature. [6], [17], [41], [61], [66], [75], [101]–[105] Given the influence that neurotransmitters and hormones have on aggression, a genetic basis of violence can be expected in related genes. [76], [106], [107] For example, the influence of serotonin transporters [18], [41], [108] and receptors, [61] tryptophan hydroxylase, [2], [77] MAO-A, [83], [106] catechol-O-methyltransferase, [18], [77], [89] dopamine receptors, [17], [41], [86]–[88], [104], [107] the androgen receptor [64], [95], [109] and the corticotrophin releasing hormone receptor [110] have been mentioned. However, in a meta-analysis including these genes, no single gene was significantly correlated with aggression. [76] Genes can still be used to have a better understanding of aggression, but not for risk assessments or to determine criminal responsibility. [76] A schematic overview of the evidence is given in table 4.

**Table 4:** Overview of the evidence for genetic influences.

Geneticinfluences	Population	Method	Outcomes	Reference
MAO-A	Subjects withchildhoodmaltreatment	Genetictesting	Correlation between low activityand antisocial behaviour	[10], [20], [76], [86], [106], [114]

For a more complete overview, see Vassos’ review [76].

Gene-gene interactions can also be expected to occur. [102], [107] Given the complex interplay of neurotransmitters, the effects of genetic polymorphisms can be corrected or aggravated by other genetic polymorphisms [102], [107].

However, aggressive behaviour is not caused by genetics alone. The ‘social push’-theory states that genes need a particular social environment to result in specific behaviour. [6] Antisocial personalities, for example, develop due to biological factors, but lead to antisocial behaviour more often if the social situation predisposes, or pushes the individual to that behaviour. [42], [56] On the other hand, if the social environment does not require antisocial behaviour to achieve what is wanted, antisocial behaviour might not develop despite an unfortunate biological background [42], [52].

This also means that the correlation between antisocial behaviour and biological risk factors becomes weaker in cases of poor social backgrounds, like a broken home. [42] This is because when the environment does not push an individual towards a negative behaviour (such as someone who has been reared in a benign social environment), but the antisocial behaviour comes to expression anyway, genetic factors have played a larger role in the instigation of the antisocial behaviour. [73] When the environment pushes too hard, like in very poor social backgrounds, every individual is influenced, resulting in a weak correlation between genetic factors and the actual behaviour [42].

To study whether it is genetic makeup or the environment that causes specific behaviour, twin and adoption studies are often used. [40], [45], [69], [82], [103], [105], [109], [111]–[113] This is because monozygotic twins share identical genetic material, and dizygotic twins share on average 50% of their dissenting genetics, [40], [82], [101], [103], [112], [114], [115], but both share an environment. [103] Subtracting the differences between these groups allows estimations of the contribution of environmental versus genetic factors when behavioural differences are measured. [101], [103], [114], [115] Twin studies have, for example, shown the relevance of both genetics and environment for the development of antisocial behaviour, violence and aggression [55], [111].

The adoption method compares the correlation between adopted children and their adopting parents with the correlation between adopted children and their biological parents. [101] This also results in an estimate of the contribution of genetic and environmental factors [101].

One of the most studied genes in research on gene-environment interactions is MAO-A (see table 4). A MAO-A deficiency has been shown to increase reactive aggression, [20], [74], [84] and its low activity increases criminal [7], [84] and antisocial behaviour. However, this last result was found especially in males when the subject had also suffered from childhood maltreatment. [10], [20], [76], [86], [106], [114] In criminal settings, those who had a promoter sequence resulting in low MAO-A activity and who had been maltreated as a child were overrepresented. [20], [116] Both examples illustrate that the effect of MAO-A is dependent on environmental factors, so the environment and genetics interact [57], [108], [116].

Gene effects rarely influence behaviour directly. MAO-A, for example, may have a role in the difference between male and female levels of violence since the MAO-A gene is encoded on the X chromosome. [7], [96], [106], [112], [113] The documented correlation between high testosterone levels and low MAO-A activity, and resulting aggression, supports the hypothesis of further testosterone-induced suppression of the MAO-A gene. [113] The promoter region of the MAO-A gene does in fact contain glucocorticoid/testosterone response elements. [20] Testosterone competes with cortisol for binding, but leads to less transcription than cortisol binding does [20].

Males have been found to have less connectivity between the orbitofrontal cortex and the amygdala, [113] lower functional connectivity between the ventromedial prefrontal cortex and the amygdala, [20] lower orbitofrontal activity [113] lower cingulate cortex activation, [113] and a larger amygdala. [117] This does not explain, however, the difference between male and female proclivity for violence. This example makes clear that the change in behaviour due to the MAO-A gene is actually a consequence of secondary effects, and cannot be explained by its direct effect on neurotransmitters alone [74].

#### Prenatal environmental factors

The prenatal period is an important time for development of the brain and influences function and the way actual behaviour is instigated later on. Exposure to several addictive substances used by the mother during this period influences brain development. [6], [32], [69] A schematic overview of the evidence is given in table 5.

**Table 5:** Overview of the evidence for involved prenatal environmental factors.

Prenatalenvironmentalfactors	Population	Method	Outcomes	Reference
Prenatal alcoholexposure	Pregnantwomen	Interview,tests	Increased risk for conduct disorder	[6]
Nicotine	Pregnantwomen	Interview,arrest history	Dose-response relationship between useduring pregnancy and violence	[6], [55]
Nutrition	Pregnantwomen	Follow-up ofoffspring	Nutritional deficits during the first twotrimesters had children with antisocialpersonality disorder more often	[32], [66]
Birthcomplication	Pregnantwomen	Follow-up ofoffspring	Anoxia, preeclampsia and forceps deliverylead to increased risk for antisocial andcriminal behaviour through braindysfunction	[6], [32]

#### Substance exposure

Prenatal alcohol exposure can cause structural deficits in the corpus callosum, [55], [67] and cerebellum in the infant. [67] It also impairs the infant’s memory [67] and executive functioning [67] and lowers IQ. [55], [67] Though the physical signs diminish in adolescence, the neuroanatomical differences remain. [67] These changes may explain why it is also found that prenatal alcohol exposure increases the risk for conduct disorder [6].

For nicotine a dose-response relationship between the number of cigarettes smoked during pregnancy and violence has been found. [6], [55] Prenatal nicotine [6], [32], [55] and carbon monoxide [32], [55] exposure is thought to disrupt the development of the noradrenergic system, [6] possibly via enhancing the muscarinic 2 (M_2_)-receptor, [32] leading to diminished sympathetic nervous system activity. This could explain the observation of low autonomic arousal in violent and antisocial individuals and criminals [6], [32].

Prenatal cocaine exposure is also associated with increased delinquency, but these results are debated [55].

#### Nutrition

Apart from addictive substances, basic nutrition during pregnancy influences the development of the baby and later behaviour. Like the well-known effects of folic acid on preventing spina bifida, other nutrients influence the development of aggressive behaviour.

Women who suffered nutritional deficits during the first and second trimester of their pregnancy gave birth to children who had antisocial personality disorder more often than the general population in two studies. [32], [66] In addition, heavy metals like copper have also been shown to influence later behaviour. High copper in the neonatal brain is associated with abnormalities in the hippocampus, [55] which is associated with violence. A low zinc to copper ratio was found in males with a history of assaultive behaviour [32].

In addition to the use of addictive substances or nutrition by a mother, birth complications are also a risk factor for prenatal development. Both of these factors could be seen as markers, although the exact mechanisms of these factors are not clear. [43] For example, a significant interaction between maternal smoking and delivery complications has been documented [43].

#### Birth complications

Birth complications such as anoxia, preeclampsia and forceps delivery lead to increased risk for antisocial and criminal behaviour through brain dysfunction. [6], [32] The hippocampus is particularly susceptible to hypoxia and anoxia. [6], [55] It is clear that birth complications interact with psychosocial risk factors, like maternal stress, poor parenting and an unstable family environment [32].

In summary, prenatal development seems to affect brain development and as such affects behaviour, which in some cases results in violent behaviour later in life. Minor physical anomalies may be considered as markers of deviant brain development during pregnancy. [6] Features like low-set ears, adherent ear lobes and a furrowed tongue are anomalies that have been described [6] and shown to predict violent offending in unstable home situations. [6], [32] A schematic overview of these factors is given in table 5.

#### Postnatal environmental factors

Although the prenatal environment has an effect on brain development, the post-natal environment also shapes brain functioning and gene expression. In the following paragraphs, several examples show how the environment may interact with genes or brain development to influence the development of aggressive or violent criminal behavior.

#### Age

An explanation for the robust observation of age as a risk factor for criminal behaviour is found in the development of the prefrontal cortex. [6], [32], [90], [109] A first explanation states that since the myelinisation of the prefrontal cortex continues into a person’s 20s or even 30****s, it simply cannot cope with the executive demands of adulthood placed upon an individual after adolescence [6], [7], [32].

A second explanation is found in the accessibility of the means, opportunity and motive for aggressive behaviour. [47], [57] During adolescence, people first experience significant physical strength and cognitive challenges, are less inhibited by supervision and experience pressure to perform both in school and relationships [6], [57], [90].

The combination of these hypotheses offers more insight. The changing environment of adolescence requires increased executive functioning, which relies on the prefrontal cortex. [6] Overload of the prefrontal cortex results in impaired development, leading to antisocial behaviour. A stable, supportive environment may offer protection from this harmful overload [6].

A third explanation is offered by the influence of testosterone. The high-risk periods of adolescence and young adulthood overlap with a testosterone curve in many cultures. [75] So the peak occurrence of violence at these ages could be caused by testosterone. [75] The peaks in sensation seeking, possibly related to testosterone and cortisol, are also seen during these time periods [90].

The highest risk of violent behaviour is indeed found in persons in their late teens and early twenties. [4], [8], [32], [57], [75], [90], [99], [118], [119] This holds for both the general population and people who are mentally ill [4].

#### Poor child-rearing

The cycle of violence hypothesis states that a history of growing up in a violent context, [73], [84] defined as a genetic predisposition, [114], [116] a history of witnessing violence or being victimized [26], [73], [88] leads to committing violence, possibly by desensitisation, and an acceptance of violence as normal. [73] This would lead to changed psychophysiological parameters like reduced cortisol and decreased autonomic arousal, possibly through an altered development of the limbic system [92].

The increased cortisol levels of infants separated from their mothers have in fact been shown. [47] This might lead to abnormalities in the hypothalamic–pituitary–adrenal axis, [10], [92] leading to hippocampal atrophy, based on stress caused by a lack of affect or traumatic childhood experiences. [92] This in turn has been hypothesised to lead to more proactive aggression [73].

This theory is supported by various backgrounds that have been found to influence or predict behaviour. For instance child abuse, [3], [4], [8], [17], [22], [26], [31], [43], [55], [57], [70], [73], [92], [112], [116], [120]–[122] witnessed violence [10], [73] or domestic violence, [3], [4], [31], [55], [57], [111], [120], [123] family criminality, [4], [45], [55], [99], [101], [120] marital conflict, [55] early puberty timing, [90], [109] early sexual activity, [85] teenage pregnancy, [55] negative emotional attitude from parents [47], [73] or mother [57], [92], [101], [112], [114], [122] and physical maltreatment [73], [111], [114], [120] are all correlated with crime and antisocial behaviour. Also, a child’s antisocial behaviour [55] or hyperactivity-impulsivity-attention deficit [55] predicts later criminal behaviour. Some of these correlations exist through direct influence, indirect influence or function as a marker [85].

As another example, sexual abuse as a child has been associated with later alcohol dependence. [124] This is a risk factor leading to violence. [124] A reduction in child abuse by 50% can be achieved by simple home visits during the first two years of child rearing. [57] Community-based programs also improve self-reported well-being of the parents. [57] Given the influence child abuse has on developing antisocial and offensive behaviour, these programs could lower crime rates.

#### Socioeconomic status

The lower socioeconomic classes, as measured by SES, are overrepresented among criminals, and a direct correlation has been found. [8], [17], [70], [78], [100], [119], [123] Sub factors of the SES classification, like poverty, [3], [17], [55] unemployment [52] and school failure [55], [99], [120], [125] have also been found to correlate with criminal behaviour. As an explanation for this finding, the increased stress caused by low economic status has been mentioned. [78], [88] In addition, serotonin response correlates with a SES-score, therefore, a lack of serotonin could confound the correlation between low SES-scores and criminality as well. [78] However, the increased need for and acceptance of violence are also mentioned. In adolescents, it was found that subjects with either high or low social status were more inclined to use physical aggression at school. Middle economic status was a protective factor [78].

#### Low IQ

Low IQ-scores, [10], [17], [57], [99], [119], [120], [125] especially for verbal intelligence, [43], [50], [99] form a risk factor for delinquency and antisocial behaviour. [10] One explanation is the expected lower achievement in school, possibly leading to exclusion, poverty and antisocial behaviour. [57] The increased risk of getting caught if one has a low IQ, or an inherent neurobiological correlate between IQ and delinquent behaviour could also account for this finding.

#### Gang membership

The association between gang membership and delinquency has been established in multiple studies, for both male and female gangs. [10], [99] Neurobehavioural deficits, such as a history of head injury or intermittent explosive disorder are found in gang members more often than in controls. [6] When corrected for other risk factors before and after membership, this association still exists. [10] It may be that individuals with neurobehavioral deficits are more likely to become a gang member (because of traits like sensation seeking) or gang membership affects brain functioning.

#### Nutritional influences

Postnatal nutritional factors and antisocial or violent behaviour are correlated. [55] For example, protein under-nutrition leads to antisocial personality disorders. [32], [55] Serotonin depletion, due to tryptophan under-nutrition (the limiting amino-acid which is used for serotonin) caused aggression under laboratory conditions, compared to well-fed controls. [2] This was also found in rats and monkeys [55].

Iron deficiency has been found in aggressive children and those with a conduct disorder. [55] In children with attention deficit-hyperactivity disorder (ADHD), both a behavioural and cognitive improvement were found when iron was supplemented. [55] High serum copper levels and high hair levels of manganese, lead and cadmium have been found in aggressive persons. [55] For some of these metals, this effect was only found in combination with low calcium levels [55].

Though not well understood, the relationship between these metals and behaviour is thought to lie in neurotransmitters. [55] The influence of metals on behaviour is debated though, since few studies have been conducted, not all results have been replicated and no prospective study or study taking other risk factors into account has been published [55].

#### Brain damage

The example of Phineas Gage is often used to illustrate the effects of brain damage. His prefrontal cortex was selectively damaged by an iron spike. [1], [3], Though he survived, his behaviour changed after the accident; he became more aggressive and socially inappropriate. [1], [3], [21], [29] Head injury is found in offenders more often than in the general population, [15], [35], [51], [100] and those with prefrontal damage exhibit aggression more often than those without [13], [44], [53].

The exact location of the injury influences the changes in behaviour. [50] Dorsal lesions lead to pseudo-depression, marked by apathy and impaired long-term planning;[37], [68] orbital lesions lead to more superficial emotional responses and pseudo-psychopathy. [8], [37], [68] Whether prefrontal cortex damage leads to criminality or socially less accepted behaviour, is not yet predictable [1].

The age of injury also has an influence. [50] When the prefrontal cortex injury occurs before adolescence, it leads to diminished executive functioning and what is called ‘acquired sociopathy’. [1] When the injury happens in adulthood, however, more impulsive and uncontrolled emotional behaviour results, but executive functioning is not reduced. [1] The age at the time of brain damage also predicts the age for the start of the criminal career of offenders [66].

However, as mentioned before, there are also case reports of people suffering from the same brain injuries as offenders, who do not show violent aggressive behaviour [21].

Overall, several environmental factors are involved in aggressive behaviour and criminality, some more understood and replicated than others. A schematic overview is given in table 6.

**Table 6:** Overview of the evidence for environmental factors.

Postnatalenvironmentalfactors	Population	Method	Outcomes	Reference
Age	Humans	Databasesearch	Highest riskfor violent behaviouris in late teens andearly twenties	[4], [8], [32],
Child abuse	Various	Various	Crime and antisocialbehaviour	[3], [4], [8], [17], [22], [26], [31], [43], [55], [57], [70], [73], [92], [112], [116], [120]–[122]
Antisocialbehaviour	Children	Follow-up	Predicts later criminalbehaviour	[55]
Hyperactivity-impulsivity-attentiondeficit	Children	Follow-up	Predicts later criminalbehaviour	[55]
Socioeconomicstatus	Humans	Various	Direct correlation withcriminality	[8], [17], [70], [78], [100], [119], [123]
Low IQ-scores	Humans	Various	Risk factor fordelinquency andantisocial behaviour	[10], [17], [57], [99], [119], [120], [125]
Gangmembership	Humans	Various	Correlation withdelinquency	[10], [99]
Irondeficiency	Aggressiveand conductdisorderedchildren,juveniledelinquents	Plasmalevels	Iron deficiency wasoverrepresented	[32], [55]

## Discussion

The aim of this paper was to review evidence of biological risk factors that predispose individuals to antisocial and violent behaviour, and to discuss their use for forensic assessment. Several aspects that complicate comparing research in this area must be mentioned to understand the usefulness of the reviewed evidence.

First, much research in this field is performed on psychiatric patients or normal populations, not on offenders. Although the number of studies using groups of offenders grew between 2000 and 2013, there is still a great need to understand specific offender subgroups. Even if studies use offenders, most groups studied fail to represent the entire imprisoned population. Different studies each select different offender groups thus making the results less valid. [50], [53], [69], [105], [120] Defining the studied population is difficult, and different choices are the cause of many differences between studies. In addition, many of the groups studied are simply too small to draw any meaningful conclusions that extend to all offenders, [3], [32], [50] or to find reliable results that can be replicated.

Second, most studies, specifically neuroimaging studies, compare groups of offenders with other groups of individuals. Forensic (risk) assessments mainly focus on a relationship between deviances and violent behaviour shown by a single individual when committing a crime. Therefore, the forensic field is in need of research showing how alterations in genes, brain, or psychophysiology influence violent behaviour in a specific individual at a specific moment in time.

Third, apart from research on gene-environment interactions, studies on the relationship between neurobiological deficits and violent behaviour that also take psychological or sociological evidence into account are scarce. Most reviewed primary research focuses on only one of the fields related to violent aggressive behaviour, not on the interaction between these fields. Violent aggression, like all forms of human behaviour, [112] does not only develop under specific genetic and environmental conditions, but rather it requires an interplay between the two. [7], [69], [76], [95], [101], [103], [108], [110], [126] Violence should be considered as the end product of a chain of events over the course of a person’s development, during which risks accumulate and potentially reinforce each other. [57] This research gap should be bridged.

Fourth, the interaction does not lead directly to violent aggressive behaviour, but to sensation seeking, impulsivity or low harm avoidance. Evidence of alterations that solely explain violent behaviour was not found. Therefore, it is unlikely that genetic or neuroscientific tools will be used as independent tests in forensic (risk) assessments.

Fifth, studies that do relate neurobiological deficits to behaviour use a variety of aggressive or antisocial behaviours that are not necessarily of use for forensic assessment, which is mainly interested in physical or violent aggression. How violence, aggression and delinquency are defined and quantified differs in every test; and self-report scales are unreliable. [96], [105], [120], [126] In addition, the distinction between violent reactive and instrumental aggression is not always clear, although these forms of violence are likely to have very disparate neurological backgrounds [2], [10], [44], [76], [101].

Sixth, different studies use a variety of techniques and methods. Neuroanatomical studies focus on imaging single subjects so it is hard to place the subject in a context where violence is likely to be triggered. Neuropsychological studies, on the other hand, often use large populations and are able to test subjects in more ecologically valid situations.

Specifically, in imaging studies, the various regions of the frontal cortex are usually not considered separately. [27], [35], [44] Also the nuclei of the amygdala are not measured separately. [54] This leads to generalisation, simplification and reduced power, since only some of these regions might actually be linked to deviant behaviour.

Also, testing levels of substances in subjects differs per study. The circadian rhythm of cortisol is not always taken into account. [72], [93] Various time periods between samples and circumstances make studies hard to compare.

To conclude, with better designed studies and more standardisation, comparing studies would be easier and it might become possible to link behaviour to underlying mechanisms [53].

## Conclusion

The influence genes and deviances in brain development have on the development of violent aggressive behaviour, and in which situation, needs further research before genetic and brain imaging information can be used in forensic assessments or in court. [11], [20], [76] Though most mechanisms are not elucidated, some of the findings may in time be used to estimate risk of recidivism in combination with psychosocial assessment tools. This means better tools for neurologically based assessment might become available as the knowledge develops.

As the developmental profile of brain areas and their vulnerabilities are being discovered, key moments to modulate specific environmental factors for persons with a high-risk genetic profile will become possible. [114] For example, some findings can be used to more accurately assess risk of criminal behaviour on an individual basis. However, there is an important ethical difference between using neurobiological assessment tools in the case of suspects and convicted offenders versus in the general population or subgroups, such as children or adolescents. Even in case of the former group, offender rights might be at stake [65].

On a more general level, knowledge of nutrition could be used to improve our society or correctional facilities, and help prevent future encounters with forensic facilities. Better guidance during the most difficult years of adolescence and home visits can diminish chances of a harmful overload of the prefrontal cortex and decrease chances of child abuse. And obviously, brain damage should be avoided. Reducing those criminogenic risk factors reduces the likelihood of engaging in criminal activity, both directly and via reduced triggering of gene-environment interactions. [103] In the future, new information from neuroscience, when integrated into the information already available from sociological and psychological assessments, could contribute to the development of better risk assessment tools, treatments and cures for offenders, reducing recidivism as well [16], [21], [63], [66].

This review underlines the importance of maintaining a case-by-case differentiated approach to evidence-based forensic assessment that takes into account the individual psychosocial development, and neurobiological and genetic risk factors contributing to violent crime.
